# Motion opponency examined throughout visual cortex with multivariate pattern analysis of fMRI data

**DOI:** 10.1002/hbm.25198

**Published:** 2020-09-02

**Authors:** Andrew E. Silva, Benjamin Thompson, Zili Liu

**Affiliations:** ^1^ School of Optometry and Vision Science University of Waterloo Waterloo Ontario Canada; ^2^ Department of Psychology University of California at Los Angeles Los Angeles California USA

**Keywords:** hMT+, motion perception, MVPA, noise reduction, V3A, V5

## Abstract

This study explores how the human brain solves the challenge of flicker noise in motion processing. Despite providing no useful directional motion information, flicker is common in the visual environment and exhibits omnidirectional motion energy which is processed by low‐level motion detectors. Models of motion processing propose a mechanism called motion opponency that reduces flicker processing. Motion opponency involves the pooling of local motion signals to calculate an overall motion direction. A neural correlate of motion opponency has been observed in human area MT+/V5, whereby stimuli with perfectly balanced motion energy constructed from dots moving in counter‐phase elicit a weaker response than nonbalanced (in‐phase) motion stimuli. Building on this previous work, we used multivariate pattern analysis to examine whether the activation patterns elicited by motion opponent stimuli resemble that elicited by flicker noise across the human visual cortex. Robust multivariate signatures of opponency were observed in V5 and in V3A. Our results support the notion that V5 is centrally involved in motion opponency and in the reduction of flicker. Furthermore, these results demonstrate the utility of multivariate analysis methods in revealing the role of additional visual areas, such as V3A, in opponency and in motion processing more generally.

## Graphical abstract

Motion opponency is the process by which opposing motion signals in the same local area are cancelled during visual motion processing, resulting in the attenuation of non‐informative flicker noise. Using multivariate pattern analysis, we examined flicker processing and motion opponency throughout human visual cortex. Our results implicate area V3A as a contributor to motion opponency and affirm the central role of V5 in the suppression of flicker noise.

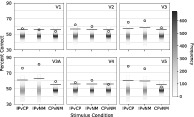

## 1 INTRODUCTION

Motion processing is an essential aspect of vision. However, the successful interpretation of directional motion information is complicated by the presence of flicker noise. Any abrupt change in the luminance of a visual scene, like a flickering light or a bright object appearing suddenly against a dark background, creates flicker noise: omnidirectional and uninformative signals which can be processed just as any true motion signal (Born & Bradley, 2005; Bradley & Goyal, 2008). Therefore, a mechanism to reduce the influence of flicker noise is essential in effective motion processing (Qian, Andersen, & Adelson, 1994).

Classic theoretical models of motion processing employ a mechanism called motion opponency to attenuate the processing of flicker. During motion opponency, a local motion output is calculated by combining all motion signals within the given local area (Adelson & Bergen, 1985; Qian et al., 1994; Reichardt, 1961; Simoncelli & Heeger, 1998; van Santen & Sperling, 1985). The omnidirectional motion signals which define flicker noise are locally balanced and therefore cancel during motion opponency. In contrast, useful motion information is typically directional and not locally balanced. As a result, motion opponency acts as a filter during motion processing, attenuating flicker information while allowing true motion signals to continue for further processing.

Physiological research has identified neural responses indicative of opponency in monkeys. Qian and Andersen (1994) designed a bidirectional and locally motion‐balanced dot stimulus in which each randomly‐positioned dot was located near a second dot traveling in the opposite direction. This stimulus is now referred to as “counter‐phase” (CP) dot motion (Lu, Qian, & Liu, 2004). Relative to a bidirectional stimulus without local motion balancing, Qian and Andersen (1994) found that MT neurons exhibited a muted response to counter‐phase stimuli. In fact, this response was not significantly greater than the MT response to flicker noise.

Neuroimaging has provided evidence for opponency in human motion processing. Reduced univariate V5 BOLD responses to counter‐phase stimuli have been reported in multiple studies and are generally consistent with Qian and Andersen's (1994) original physiological work (Heeger, Boynton, Demb, Seidemann, & Newsome, 1999; Muckli, Singer, Zanella, & Goebel, 2002; Thompson, Tjan, & Liu, 2013). However, suggestions exist that motion opponency and local directional pooling may be distributed throughout the visual cortex in humans (Garcia & Grossman, 2009). Consistent with a multi‐region network of local motion pooling, Huck and Heeger (2002) found that relatively high pattern motion‐selective responses, indicative of local motion integration, were not exclusive to V5, occurring also in areas V2 and above.

Various nonopponent stimuli have been employed as a comparison against the counter‐phase stimulus. Often, a stimulus containing the same bidirectional local signals, but without local balancing is employed. One such example of a bidirectional and nonopponent stimulus has been referred to as “in‐phase” (IP) (Lu et al., 2004; Silva & Liu, 2015; Silva & Liu, 2018; Thompson et al., 2013). The in‐phase (IP) stimulus is nearly identical to a counter‐phase (CP) stimulus, except that both dots within a pair travel in the same direction.

While previous research may be consistent with opponency in the human brain, the human brain's responses to counter‐phase and flicker stimuli have never been directly compared. Because the theoretical formulation of motion opponency selectively reduces flicker noise processing, a more complete understanding of motion opponency in the human brain may be achieved by examining the suppressed response to flicker noise (Adelson & Bergen, 1985; Qian et al., 1994; Reichardt, 1961; Simoncelli & Heeger, 1998; van Santen & Sperling, 1985). If the reduced BOLD response to motion opponent stimuli reported in previous human studies is indeed analogous to theoretical motion opponency, then the human brain may process counter‐phase and flicker stimuli similarly. Because both flicker and counter‐phase motion stimuli exhibit locally balanced motion, a motion opponent system should output zero net motion in both cases.

With the emergence of multivariate pattern analysis (MVPA) as a powerful tool for understanding neural processing using fMRI (Mahmoudi, Takerkart, Regragui, Boussaoud, & Brovelli, 2012; Norman, Polyn, Detre, & Haxby, 2006; Tong & Pratte, 2012), a detailed exploration of flicker and counter‐phase motion processing is now possible. The traditional fMRI region‐of‐interest analysis involves averaging the responses of all voxels with a region to calculate a single averaged univariate BOLD response. In MVPA classification, a region‐wide and voxel‐level pattern of activation is inputted, and a classification algorithm predicts which stimulus likely elicited the given brain response. This allows a comparison between stimuli that elicit the same univariate response despite potentially eliciting different patterns of voxel activations.

The current study is the first to examine flicker processing and motion opponency by applying multivariate analysis techniques to fMRI data. Our primary analysis focused on V5. We trained multivariate classifiers with BOLD data associated with in‐phase (IP) stimuli, counter‐phase (CP) stimuli, or nonmotion (NM) stimuli exhibiting incoherent onset and offset flicker but no smooth translational movement. Classifiers were trained to discriminate two of the three different stimuli and tested on both trained and untrained stimuli. We predicted the following pattern of results:

1. The multivariate classifier will correctly discriminate IP stimuli from CP and NM stimuli. This result would be consistent with motion opponent processing.
2. The multivariate classifier will systematically misclassify CP stimuli as NM. This result would be consistent with CP stimuli eliciting a similar neural representation to NM due to motion opponency.
3. The multivariate classifier will systematically misclassify NM stimuli as CP. This result would also be consistent with CP stimuli eliciting a similar neural representation to NM due to motion opponency.

As a secondary analysis, we explored the performance of classifiers trained using BOLD data from V1, V2, V3, V3A, and V4 to assess whether BOLD responses indicative of opponency were present throughout the human visual system.

## 2 MATERIALS AND METHODS

### 2.1 Apparatus, stimuli, and experimental procedure

All experimental stimuli were programmed in Python using the Psychopy library (Peirce, 2007; Peirce, 2009). Stimuli were back‐projected onto a screen (12 cm × 9 cm useable area, 1,024 × 768 resolution, 60 Hz refresh rate) that was mounted above the fMRI head coil. Participants viewed the display through a mirror. Due to differences in head size, viewing distances ranged between 22 and 25 cm, and therefore the size of one pixel ranged between 0.027 and 0.031°. All stimuli were presented in front of a solid gray background (luminance 4 cd/m^2^).

Participants fixated on a central black square dot 5 pixels in size throughout an entire experimental run. The visual presentation alternated between a 12‐s stimulus block and a 12‐s blank block displaying only the fixation point. During the stimulus blocks, 250 pairs of randomly distributed white square dots (luminance 67 cd/m^2^) of size 3 pixels were presented. Each dot was initially placed no more than 8 pixels away from its paired partner along a common orientation, creating a Glass pattern (Glass, 1969). The Glass pattern could be oriented either horizontally or vertically in any given block. All dots had a limited dot lifetime of 150 ms before being randomly replotted.

To engage attention, a mildly effortful behavioral task was employed. Stimulus blocks were divided into 6 trials, each lasting 1.1 s. On each trial, the Glass pattern orientation was 15° clockwise or counterclockwise from the block's overall cardinal orientation. Each block contained three clockwise and three counterclockwise trials. Participants indicated the orientation of each trial using a button response box. All participants achieved ceiling performance. An inter‐trial interval of 500 ms was used, during which no dots were presented.

Three different paired‐dot stimulus conditions were presented separately in blocks that were randomly interleaved throughout each scanning run. Each block could be composed of counter‐phase (CP), nonmotion (NM), or in‐phase (IP) stimuli. During CP blocks, the two dots in a pair traveled in opposite directions. CP pairs were initially separated by 8 pixels along the Glass pattern orientation and traveled toward one another, crossed, and were randomly replotted after again achieving a separation of 8 pixels. To temporally stagger the replotting of CP dots, each CP pair was initially plotted at a randomly selected point along its full trajectory.

During IP blocks, both dots within a pair traveled in the same direction along the orientation of the Glass pattern. Each pair was independently assigned a random initial lifetime to temporally stagger the replotting of dot pairs, and each pair was independently assigned a random within‐pair distance between 0 and 8 pixels. Different IP pairs traveled in opposite directions along the Glass pattern orientation, creating a bidirectional stimulus. Because IP and CP dots all traveled 8 pixels during their 150 ms limited lifetime, the dot speed ranged from 2.3 to 2.6°/s, depending on viewing distance.

**FIGURE 1:**
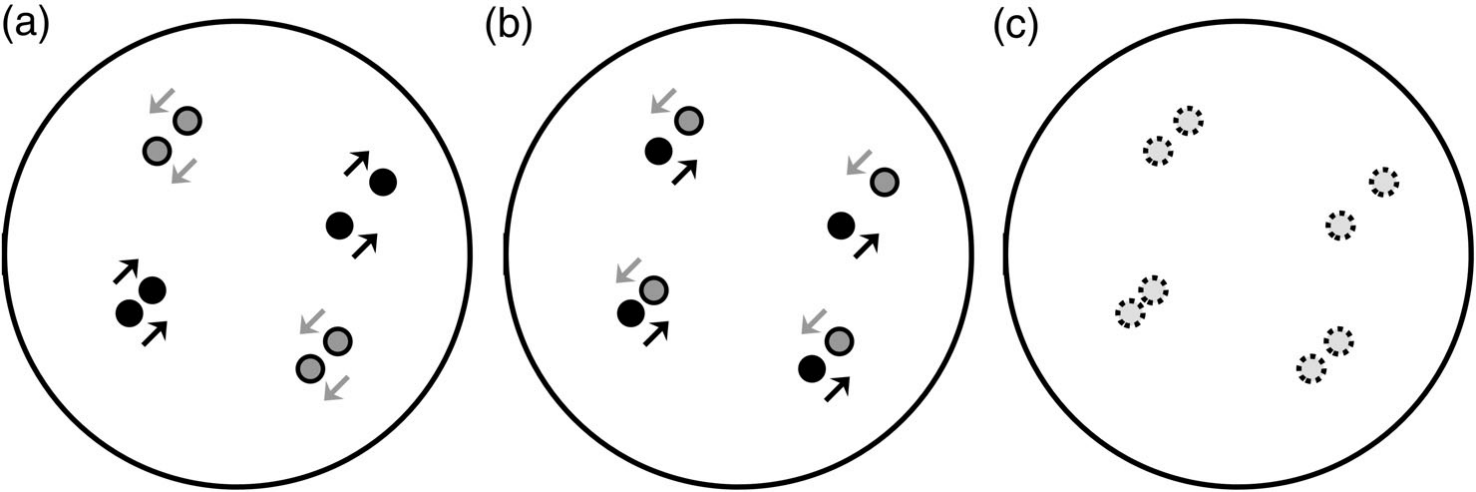
Diagrams of in‐phase (a), counter‐phase (b), and nonmotion stimuli (c). Dots are shaded according to their direction of motion. Nonmotion dots are represented by broken circles. All dots had limited lifetimes and the stimuli exhibited indistinguishable Glass patterns

NM dots behaved identically to in‐phase dots, except that there was no translational motion. Critically, in‐phase and counter‐phase stimuli contained the same number of left and right motion signals, and the Glass patterns of all three conditions were indistinguishable from one another. One experimental run contained 6 blocks of each paired‐dot condition, totaling 18 blocks per run. Each participant performed 8 runs, totaling 144 blocks (48 blocks per condition). Stimulus diagrams are presented in Figure 1. See Supporting Information Video A for examples of the IP, CP, and NM stimuli and the behavioral task.

### 2.2 Participants

Functional neuroimaging data were collected from five participants. All participants had normal or corrected‐to‐normal vision. Informed consent was obtained, and all participants were treated in accordance with the Code of Ethics of the World Medical Association (Declaration of Helsinki). For their participant, participants received CAN$200 (CAN$50 per scanning hour).

### 2.3 Magnetic resonance imaging

All scans took place in the Centre for Functional and Metabolic Mapping at the University of Western Ontario's Robarts Research Institute on the 7T Siemens Magnetom scanner. All functional scans used an 8‐channel transmit, 32‐channel receive coil optimized for the occipital pole and providing an unobstructed field‐of‐view to the visual stimulus. All anatomical scans used an 8‐channel transmit, 32‐channel whole‐head coil. For each participant, we collected an anatomical scan (MP2RAGE, 224 sagittal slices, 0.7 mm isotropic voxel size, TR = 6,000 ms, TE = 2.73 ms, Flip angle 1 = 4°, Flip angle 2 = 5°, TI 1 = 800 ms, TI 2 = 2,700 ms), two retinotopy scans, one with rotating wedge stimuli and one with expanding ring stimuli (60 coronal slices originating at the posterior pole, 1.5 mm isotropic voxel size, TR = 1,000 ms, TE = 19.6 ms, Flip angle = 45°, slice order: interleaved, phase condition direction: FH, pulse: gradient echo, imaging type: EPI), one V5 localizer scan (60 coronal slices originating at the posterior pole, 1.5 mm isotropic voxel size, TR = 1,600 ms, TE = 19.6 ms, Flip angle = 45°, slice order: interleaved, phase condition direction: FH, pulse: gradient echo, imaging type: EPI), and eight experimental scans (60 coronal slices originating at the posterior pole, 1.5 mm isotropic voxel size, TR = 1,200 ms, TE = 19.6 ms, Flip angle = 45°, slice order: interleaved, phase condition direction: FH, pulse: gradient echo, imaging type: EPI).

### 2.4 Preprocessing

The fMRI data preprocessing, the functional localizer analysis, and the univariate experimental analysis were conducted using BrainVoyager QX 2.8.4 (Formisano, Di Salle, & Goebel, 2006; Goebel, Esposito, & Formisano, 2006). Functional data were preprocessed using motion correction, slice scan time correction, and highpass filtering. The functional scans were coregistered to the anatomical scan, and both scans were brought to Talairach space for region of interest functional localization using BrainVoyager's coregistration and visualization tools. The ROIs were transformed back to native functional space for multivariate analyses using the MATLAB toolbox NeuroElf (www.neuroelf.net). All transformations were applied with sinc interpolation.

### 2.5 Regions of interest localization

A standard rotating wedge and expanding ring retinotopic mapping procedure was used to identify areas V1, V2, V3, V3A, and V4 (Engel, Glover, & Wandell, 1997; Sereno et al., 1995). The black‐and‐white checkerboard wedges spanned 45°, shifted 11.25° per TR (1,000 ms) and completed seven full cycles during the session. The checkerboard rings began centrally and expanded into the periphery once per TR (1,000 ms). Twenty such expansions per cycle occurred, and seven full cycles were completed during the session. The largest ring had an outer radius of 384 pixels (between 10.4° and 11.9°) and an inner radius of 270 pixels (between 7.3° and 8.4°). The retinotopic stimuli flickered and reversed their contrast polarity at a rate of 8 Hz. V5 localization stimuli were composed of 1,348 white square dots with a side length of three pixels alternating between inward and outward radial motion. The dots traveled four pixels per frame (between 6.5 and 7.4°/s) and reversed direction every 2 s. Four 16‐second blocks were presented, alternating with 16‐second blank periods containing completely static dots exhibiting no limited lifetime. In every localization scan, participants performed a fixation task, indicating when the central fixation randomly alternated between “O” and “X.”

Bilateral V5 was identified for each participant. First, a GLM was fit to the V5 localization data using a box‐car stimulus model and BrainVoyager's default double‐gamma HRF. The model additionally contained *z*‐scored head‐motion nuisance regressors. A whole‐brain, voxel‐wise contrast of moving dots verses static dots was applied (FDR, *q* < 0.05). V5 was defined as significant clusters of voxels bilaterally located near the ascending limb, or the posterior continuation, of the inferior temporal sulcus or the posterior bank of the superior temporal sulcus (Dumoulin et al., 2000).

To identify areas V1–V4, a 3D brain surface model was constructed from the skull‐stripped and Talairach‐transformed anatomical scan in BrainVoyager. The surface was inflated, cut across the calcarine sulcus, flattened, and corrected for surface distortions. A whole‐brain, voxel‐wise cross‐correlation analysis was carried out and mapped onto the flattened brain surface, and the borders of V1–V4 were identified by observing the cross‐correlation polarity reversals running along the calcarine sulcus. One GLM was fit using all experimental data collected for the participant with one regressor for each stimulus condition and *z*‐scored head‐motion nuisance regressors. A voxel‐wise contrast of stimulus period verses blank period was applied (FDR, *q* < 0.05). The final ROIs were defined as the significant voxels within the ROI borders.

### 2.6 fMRI analysis

For visualization, model‐independent univariate BOLD time courses were extracted from the Talairach‐space transformed data for each stimulus condition and visual area. However, the data were transformed to native functional space for the main MPVA analysis. Within‐subject multivariate pattern classification analyses were carried out on fitted voxel‐wise GLM betas using support vector machines for each visual area. The GLMs for multivariate pattern classification were calculated using NeuroElf and were separate from the GLMs for ROI localization. One GLM per ROI was fitted with each individual block as a separate regressor (Mumford, Turner, Ashby, & Poldrack, 2012; Rissman, Gazzaley, & D'Esposito, 2004) and with *z*‐scored head‐motion data as additional nuisance regressors. Each block was modeled as an individual box‐car, convolved with NeuroElf's default double‐gamma HRF and *z*‐score normalized.

The classification analysis utilized Linear Support Vector Machines programmed in Python with the Scikit‐learn library using the default hyperparameter settings (Pedregosa et al., 2011). Three independent classifiers were trained to discriminate between, and then tested on, IP and CP, IP and NM, and CP and NM blocks. All SVMs utilized eightfold cross validation, whereby the classifier was trained on 7 of 8 runs and tested on the remaining run. This occurred eight times per SVM such that each run, in turn, served as the testing set, and the final performance was the average of all eight folds. Data from all identified ROIs were analyzed in this way.

A further classification analysis was carried out on data from visual area ROIs exhibiting greater than 70% group‐mean accuracy in at least two of the three pairwise classifiers. Three pairwise SVMs were trained identically as in the previous analysis, but the testing dataset was composed of data from the untrained condition. For example, the classifier trained on seven folds of IP and CP data was tested on the NM data of the eighth fold, and an eight‐fold cross validation scheme was again used. This analysis was used to probe for the presence of any systematic misclassification bias between in‐phase, counter‐phase, and nonmotion conditions. Because this analysis is unlikely to uncover systematic classification bias if the previous condition discrimination analysis performs poorly, it was only carried out with data from ROIs exceeding 70% classification accuracy in two condition discrimination classifiers.

In all multivariate analyses, significance was established using within‐subject permutation tests in which the condition labels were randomly permuted within runs, ensuring that each run preserved the same number of each type of label (Etzel & Braver, 2013). A total of 15,200 permuted datasets were tested, the maximum number of permutations our computing technology reasonably allowed. All participants received the same permutations, and the results of each permutation were averaged across participants to create one group null distribution against which the group average performance could be compared (Etzel, 2015). The permuted *p* was defined as the percentile rank of the nonpermuted group average divided by the total number of permutations +1 (15,201).

## 3 RESULTS

### 3.1 Univariate results

**FIGURE 2:**
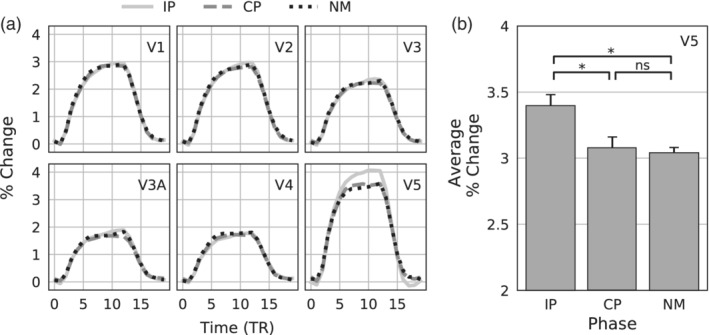
Univariate data. (a) Group‐averaged univariate time‐series during IP, CP, and NM blocks plotted as percent change for all ROIs. Data were normalized relative to the onset of the visual stimulus (TR = 0). (b) Average % change in V5. Error bars are ±1 within‐subject standard errors (Cousineau, 2005). Asterisks denote significance, *p* < .05

Figure 2a shows the average BOLD time‐series of IP, CP, and NM blocks, collapsed across participants and percent‐normalized by the voxel intensity of the first TR at stimulus onset. Consistent with previous studies, visual inspection reveals an increased IP BOLD response at area V5 and mostly overlapping activity across all three conditions for the other visual areas tested.

To test for significance in the V5 timeseries, an average % change was calculated separately for each participant and stimulus condition by averaging the values of 10 successive TRs (12 s, the length of one block) in the time series, beginning with the fourth TR (4.8 s after stimulus onset) to account for the hemodynamic response delay. The average IP, CP, and NM values were tested using a one‐way repeated‐measures ANOVA, finding a significant effect of stimulus condition *F*(2,8) = 5.1, *p* = .038. Pairwise contrasts found a significant difference between IP (3.39) and CP (3.08), *t*(8) = 2.6, *p* = .032, and between IP and NM (3.04), *t*(2,8) = 2.9, *p* = .020. There was no significant difference between CP and NM. Figure 2b plots the stimulus averages.

### 3.2 Multivariate results

For all MVPA analyses, significance was determined using a permutation test with 15,200 random permutations. Therefore, the minimum *p* possible is $\frac{1}{15,201}=6.6\times 10^{-5}$ when the true value is more extreme than every null value. IP, CP, and NM blocks were used to train and test IP v. CP, IP v. NM, and CP v. NM classifiers to examine the separability of each condition with a one‐tailed permutation test. A Bonferroni correction for multiple comparisons was applied. This analysis contains three comparisons across six ROIs; therefore, a critical *p* of $\frac{0.05}{18}=2.8\times 10^{-3}$ was set to determine better‐than‐chance accuracy. See Supporting Information Table A (discrimination performance) and Table B (misclassification bias) for summary results within all ROIs.

**FIGURE 3:**
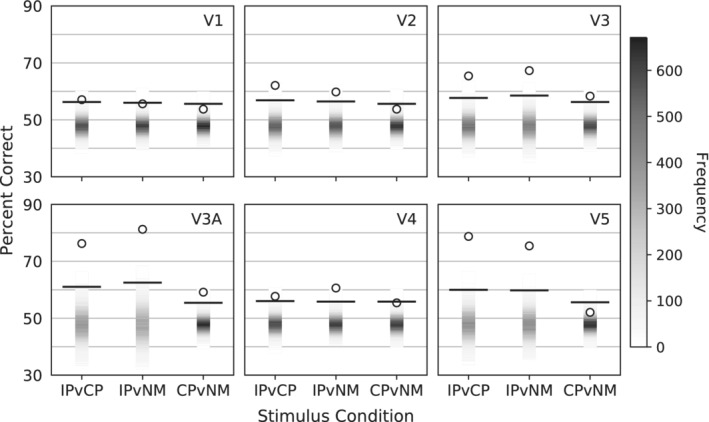
Condition discrimination MVPA results plotted in percent correct. IPvCP plots the discrimination between in‐phase and counter‐phase. IPvNM plots the discrimination between in‐phase and nonmotion. CPvNM plots the discrimination between counter‐phase and nonmotion. The shaded bar represents the estimated null distribution. Darker shades represent a higher frequency of values achieving the associated percent correct. The null distribution was estimated using 15,200 permutations. Performance using the unpermuted dataset is plotted as circles. The lines illustrate the performance required to exceed the critical *p* of 2.8 × 10^−3^

Every ROI achieved greater than chance performance when discriminating IP and CP. In increasing performance order: V1—57%, *p* = 9.2 × 10^−4^; V4—58%, *p* = 3.3 × 10^−4^; V2—62%, *p* = 6.6 × 10^−5^; V3—65%, *p* = 6.6 × 10^−5^; V3A—76%, *p* = 6.6 × 10^−5^; V5—79%, *p* = 6.6 × 10^−5^. Area V1 failed to achieve the significance cutoff when discriminating IP and NM: 56%, *p* = 4.4 × 10^−3^. However, all other areas successfully discriminated IP and NM. In increasing performance order: V2—60%, *p* = 1.3 × 10^−4^; V4—61%, *p* = 6.6 × 10^−5^; V3—67%, *p* = 6.6 × 10^−5^; V5—75%, *p* = 6.6 × 10^−5^; V3A—81%, *p* = 6.6 × 10^−5^. When discriminating CP and NM, performance was relatively poorer across all ROIs. Only data from areas V3 and V3A surpassed the threshold of significance, V3–58%, *p* = 6.6 × 10^−5^; V3A—59%, *p* = 6.6 × 10^−5^. The remaining ROIs did not achieve significance when discriminating CP and NM, V5–52%, *p* = 1.5 × 10^−1^; V1–54%, *p* = 3.0 × 10^−2^; V2–54%, *p* = 3.5 × 10^−2^; V4–55%, *p* = 4.5 × 10^−3^. The condition discrimination results for all ROIs are plotted in Figure 3.

Individual‐subject discrimination was also examined to assess whether individual‐subject trends were consistent with the group analysis. Areas V1–V4 did not exhibit consistent trends across participants. See Supporting Information Table C for these results. At V3A and V5, the individual‐subject data largely supported the main group analysis. The classifier could not discriminate NM from CP with any single‐subject dataset at area V5, but the classifier was able to significantly discriminate IP from both CP and NM: lowest accuracy 69%, *p* = 5.3 × 10^−4^ with every single‐subject dataset. The results from Area V3A were similar, though less consistent, with one participant dataset only eliciting uncorrected significance when discriminating IP and CP, 61%, *p* = .026 and mixed results when discriminating NM from CP. Table 1 presents all individual‐subject results.

**TABLE 1:** V3A and V5 individual‐subject discrimination results and significance

		IPvCP		IPvNM		CPvNM	
	Participant	%	*p*	%	*p*	*%*	*p*
V3A	0	86	6.6E – 05^a^	90	6.6E − 05^a^	66	1.1E − 03^a^
	1	89	6.6E − 05^a^	92	6.6E − 05^a^	67	1.3E − 04^a^
	2	61	2.6E – 02^b^	68	3.3E − 04^a^	51	4.3E − 01
	3	70	1.3E − 04^a^	74	6.6E − 05^a^	55	1.4E − 01
	4	75	1.3E − 04^a^	83	6.6E − 05^a^	57	3.5E − 02^b^
V5	0	89	6.6E − 05^a^	77	6.6E − 05^a^	54	2.0E − 01
	1	70	1.3E − 04^a^	79	6.6E − 05^a^	54	1.8E − 01
	2	80	6.6E − 05^a^	79	6.6E − 05^a^	49	6.2E − 01
	3	81	6.6E − 05^a^	73	6.6E − 05^a^	53	2.5E − 01
	4	74	6.6E − 05^a^	69	5.3E − 04^a^	50	5.6E − 01

Abbreviations: CP, counter‐phase stimulus. IP, in‐phase stimulus; NM, nonmotion stimulus.

^a^ Significance at Bonferroni‐corrected *p* = 2.8E − 03.

^b^ Significance at uncorrected *p* = .05.

Because the group data from V3A and V5 achieved at least 70% accuracy in two comparisons, these data were submitted to a further analysis designed to probe for any systematic misclassification biases. Three classifiers were trained to discriminate between IP and CP, IP and NM, and CP and NM identically as in the previous analysis, but they were tested with the untrained condition to determine whether the untrained condition would elicit a consistent misclassification error. A two‐tailed permutation test was used to determine significance, and a Bonferoni correction for multiple comparisons was applied. This analysis contains three comparisons across two ROIs; therefore, a critical *p* of $\frac{0.05}{6}=8.3\times 10^{-3}$ was set to determine better‐than‐chance performance.

**FIGURE 4:**
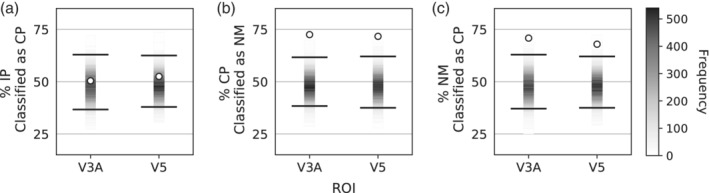
Misclassification bias analysis. (a). Results of the classifier trained to discriminate CP and NM and tested with NM data, plotted as the percent of NM blocks misclassified as CP. (b). Results of the classifier trained to discriminate IP and NM and tested with CP data, plotted as the percent of CP blocks misclassified as NM. (c). Results of the classifier trained to discriminate IP and CP and tested with NM data, plotted as the percent of NM blocks misclassified as CP. Plotting conventions are identical to Figure 2. Because this analysis involves six comparisons, the lines illustrate the performance required to exceed the critical *p* of 8.3 × 10^−3^

The classifier trained to discriminate CP and NM was tested with IP data, and there was no systematic misclassification of IP in either V3A or V5 (see Figure 4a). However, a significant misclassification bias was found with the classifier trained to discriminate IP and NM and tested with CP data. CP data was classified more often as NM in both ROIs; V3A—73% classified as NM, *p* = 6.6 × 10^−5^; and V5—72% classified as NM, *p* = 6.6 × 10^−5^ (See Figure 4b). Similarly, a significant misclassification bias was found with the classifier trained to discriminate IP and CP and tested with NM data. NM data was classified more often as CP in both ROIs; V3A—71% classified as CP, *p* = 1.3 × 10^−4^; and V5—68% classified as CP, *p* = 3.9 × 10^−4^ (See Figure 4c).

Individual‐subject misclassification analyses were examined in areas V3A and V5. Four out of five individual‐subject datasets elicited 70% performance in at least two discrimination tasks in both V3A and V5. These datasets individually achieved the group‐level performance cutoff and will be discussed as “high performing datasets.” It should be noted that participant identities differed between the V3A and V5 high performing datasets. Table 2 presents all individual‐subject misclassification bias results.

**TABLE 2:** V3A and V5 individual‐subject misclassification bias results and significance

		IP		CP		NM	
	Participant	% as CP	*p*	% as NM	*p*	% as CP	*p*
V3A	^a^0	42	5.4E − 01	77	6.7E − 03^b^	77	1.3E − 02^c^
	^a^1	23	5.3E − 02	85	2.6E − 04^b^	90	6.6E − 05^b^
	2	67	2.1E − 01	73	2.3E − 02^c^	46	7.1E − 01
	^a^3	60	5.2E − 01	60	3.8E − 01	77	1.6E − 02^c^
	^a^4	60	5.6E − 01	67	1.4E − 01	65	2.3E − 01
V5	^a^0	54	8.2E − 01	88	4.6E − 04^b^	77	3.8E − 03^b^
	^a^1	33	1.6E − 01	60	2.7E − 01	77	9.9E − 04^b^
	^a^2	67	2.0E − 01	85	6.6E − 04^b^	65	1.4E − 01
	^a^3	52	8.8E − 01	67	1.8E − 01	73	4.5E − 02^c^
	4	56	7.0E − 01	58	4.3E − 01	48	9.2E − 01

Abbreviations: CP, counter‐phase stimulus; IP, in‐phase stimulus; NM, nonmotion stimulus.

^a^ High performing dataset.

^b^ Significance at Bonferroni‐corrected *p* = 8.3E − 03.

^c^ Significance at uncorrected *p* = .05.

At Area V3A, all five individual‐subject datasets misclassified CP as NM more often than IP, and two datasets individually reached Bonferroni corrected statistical significance. The four high performing datasets also misclassified NM as CP more often than IP, with one reaching the Bonferroni significance cutoff and two reaching uncorrected significance (*p* < .05). No dataset demonstrated IP misclassification bias.

At area V5, all five datasets misclassified CP as NM more often than IP, with two datasets achieving Bonferroni‐corrected statistical significance. The four high performing datasets also misclassified NM as CP more than IP, with two achieving Bonferroni‐corrected statistical significance and a third reaching uncorrected significance. No dataset demonstrated IP misclassification bias.

## 4 GENERAL DISCUSSION

The current study examined the human motion opponency system using a novel nonmotion flicker‐based stimulus and a multivariate analysis of fMRI data. Motion opponency involves the pooling of local motion signals to output an overall motion direction and is therefore useful in flicker noise reduction (Adelson & Bergen, 1985; Qian et al., 1994; Reichardt, 1961; Simoncelli & Heeger, 1998; van Santen & Sperling, 1985). As a result, a motion‐opponent system may process counter‐phase motion and flicker noise similarly. We therefore hypothesized that BOLD data from any visual area involved in opponency would elicit a specific multivariate signature: (1) strong separability of in‐phase data and (2) systematic misclassification of counter‐phase blocks as nonmotion and nonmotion blocks as counter‐phase.

Previous neuroimaging work reported suppressed univariate counter‐phase V5 responses (Garcia & Grossman, 2009; Heeger et al., 1999; Muckli et al., 2002; Thompson et al., 2013). The current study directly extended this result by comparing the counter‐phase and nonmotion flicker responses to each other as well as to the in‐phase response. Our multivariate predictions were fully born out at the group level within V3A and V5. These results are consistent with the notion that V5 similarly processes counter‐phase and flicker stimuli and that motion opponent suppression is recruited during the processing of both stimuli.

Motion opponency is typically associated with area V5/MT receiving inputs from V1 (Bradley & Goyal, 2008; Qian & Andersen, 1994). However, the present results also suggest involvement of V3A in motion opponency, finding robust multivariate signals of opponency in V3A. However, the current study found different result profiles between these areas. Unlike area V5, the V3A univariate in‐phase, counter‐phase, and nonmotion timeseries curves appear to overlap (See Figure 2). One possible explanation for the univariate time‐series overlap of IP, CP, and NM blocks is that the population of V3A neurons participating in opponency is too small to be visually apparent in the univariate BOLD response. Multivariate classification methods are more powerful than univariate methods (Mur, Bandettini, & Kriegeskorte, 2009; Tong & Pratte, 2012), and the presence of motion opponency throughout the visual system might be more reliably detected with these more powerful methods.

Furthermore, even while both V3A and V5 exhibited significant misclassification bias between CP and NM blocks, the group V3A data exhibited above‐chance discrimination of CP and NM, unlike the V5 dataset. Therefore, these results support the notion that while V5 occupies a central role in motion opponency and cannot distinguish between counter‐phase motion and nonmotion flicker, area V3A also potentially contributes to a motion opponency network while still processing counter‐phase stimuli as motion.

Because opponency contributes a necessary noise‐reduction step in motion processing, the suggestion that V3A participates in opponency is consistent with previous findings that V3A participates in motion processing and cooperates with V5 in motion perceptual learning (Braddick et al., 2001; Chen et al., 2015; Chen, Cai, Zhou, Thompson, & Fang, 2016; Tootell et al., 1997). Interestingly, we observed significant IP discrimination against both CP and NM at V2–V4, potentially consistent with the previous findings of local motion integration across the whole visual cortex (Garcia & Grossman, 2009; Huk & Heeger, 2002). However, these results were not as consistent across individual‐subject analyses, nor did their overall discrimination performance achieve the 70% cutoff achieved by V3A and V5. Therefore, with the limited power afforded by the current study's small sample size, no conclusions about whether these additional extrastriate areas meaningfully contribute to a global motion opponency network can be made on the basis of the current study alone.

The results of the current study strengthen the idea that both counter‐phase and flicker stimuli elicit motion opponency in the human brain. Furthermore, they demonstrate that multivariate analyses are powerful tools to examine motion opponency throughout the visual system, providing evidence that area V3A may participate in motion opponency alongside V5. Further work is required to clarify V3A's potential role in motion opponency and to examine the full motion opponent network in the human brain.

## CONFLICT OF INTEREST

The authors have no conflict of interest to declare.

## DATA AVAILABILITY STATEMENT

The data that support the findings of this study are available from the corresponding author upon reasonable request.

## ETHICS STATEMENT

The authors obtained the appropriate ethics approval from the UCLA Institutional Review Board as well as the University of Waterloo Office of Research Ethics to perform this research.

## PATIENT CONSENT STATEMENT

No medical patients were recruited for this study. Nevertheless, Informed consent was obtained from all participants, and all participants were treated in accordance with the Code of Ethics of the World Medical Association (Declaration of Helsinki).

## Supporting information


**Table A** Group‐level discrimination performance across all ROIs.
**Table B:**Group‐level misclassification bias across all ROIs.
**Table C:** Individual‐subject discrimination performance across all ROIs.

**Video A** Example video of IP, CP, and NM trials composing a full experimental stimulus block. During the experimental scanning sessions, a block contained six trials of the same paired‐dot stimulus condition, all exhibiting Glass patterns that were randomly oriented 15° clockwise or counterclockwise from the block's overall cardinal direction. Trials were 1.1 s long, and participants indicated whether the Glass pattern was clockwise or counterclockwise. In the video, three vertical IP trials are presented, oriented counterclockwise, counterclockwise, and clockwise, respectively. Three vertical CP trials are then presented, also oriented counterclockwise, counterclockwise, and clockwise, respectively. Finally, three vertical NM trials are presented, oriented clockwise, counterclockwise, and counterclockwise.
