# Supplementary material for: Motion opponency examined throughout visual cortex with multivariate pattern analysis of fMRI data
Source: Hum Brain Mapp. 2020 Sep 2;42(1):5–13. doi: 10.1002/hbm.25198 (PMC7721233; doi:10.1002/hbm.25198)
Supplement: Supplementary file 1 — Table A Group‐level discrimination performance across all ROIs. Table B:Group‐level misclassification bias across all ROIs. Table C: Individual‐subject discrimination performance across all ROIs. [file HBM-42-5-s001.pdf]

**Table A:** Group-level discrimination performance across all ROIs.

| | **IPvCP** | | **IPvNM** | | **CPvNM** | |
|---|---|---|---|---|---|---|
| | **%** | ***p*** | **%** | ***p*** | **%** | ***p*** |
| **V1** | 57 | 9.2E-04** | 56 | 4.4E-03* | 54 | 3.1E-02* |
| **V2** | 62 | 6.6E-05** | 60 | 1.3E-04** | 54 | 3.6E-02* |
| **V3** | 65 | 6.6E-05** | 67 | 6.6E-05** | 58 | 6.6E-05** |
| **V3A** | 76 | 6.6E-05** | 81 | 6.6E-05** | 59 | 6.6E-05** |
| **V4** | 58 | 3.3E-04** | 61 | 6.6E-05** | 55 | 4.5E-03* |
| **V5** | 79 | 6.6E-05** | 75 | 6.6E-05** | 52 | 1.5E-01 |

* denotes significance at uncorrected *p* = 0.05.
** denotes significance at Bonferoni-corrected *p* = 2.8E-03 from main text.
IP: In-phase stimulus. CP: Counter-phase stimulus. NM: Non-motion stimulus.

**Table B:** Group-level misclassification bias across all ROIs.

| | **IP** | | **CP** | | **NM** | |
|---|---|---|---|---|---|---|
| | **% as CP** | ***p*** | **% as NM** | ***p*** | **% as CP** | ***p*** |
| **V1** | 63 | 8.9E-03* | 55 | 2.4E-01 | 46 | 3.9E-01 |
| **V2** | 64 | 4.9E-03** | 58 | 9.3E-02 | 49 | 8.9E-01 |
| **V3** | 53 | 6.8E-01 | 59 | 4.2E-02* | 58 | 1.3E-01 |
| **V3A** | 50 | 9.6E-01 | 73 | 6.6E-05** | 71 | 1.3E-04** |
| **V4** | 53 | 5.9E-01 | 57 | 1.5E-01 | 59 | 4.6E-02* |
| **V5** | 53 | 6.5E-01 | 72 | 6.6E-05** | 68 | 3.9E-04** |

* denotes significance at uncorrected *p* = 0.05.
** denotes significance at Bonferoni-corrected *p* = 8.3E-03 from main text.
IP: In-phase stimulus. CP: Counter-phase stimulus. NM: Non-motion stimulus.

**Table C:** Individual-subject discrimination performance across all ROIs.

| | **Participant** | **IPvCP** % | *p* | **IPvNM** % | *p* | **CPnVM** % | *p* |
|---|---|---|---|---|---|---|---|
| | **0** | 68 | 3.3E-04** | 57 | 7.2E-02 | 64 | 4.1E-03* |
| | **1** | 53 | 3.0E-01 | 53 | 2.7E-01 | 45 | 8.9E-01 |
| **V1** | **2** | 53 | 2.9E-01 | 54 | 1.8E-01 | 55 | 1.3E-01 |
| | **3** | 52 | 3.3E-01 | 59 | 2.2E-02* | 48 | 7.0E-01 |
| | **4** | 59 | 1.9E-02* | 54 | 1.9E-01 | 57 | 3.6E-02* |
| | **0** | 75 | 6.6E-05** | 74 | 6.6E-05** | 60 | 2.3E-02* |
| | **1** | 55 | 1.7E-01 | 58 | 3.8E-02* | 47 | 7.8E-01 |
| **V2** | **2** | 60 | 2.4E-02* | 52 | 3.3E-01 | 55 | 1.7E-01 |
| | **3** | 56 | 9.9E-02 | 52 | 3.3E-01 | 53 | 2.5E-01 |
| | **4** | 64 | 1.4E-03** | 63 | 2.8E-03** | 53 | 2.5E-01 |
| | **0** | 82 | 6.6E-05** | 80 | 6.6E-05** | 78 | 6.6E-05** |
| | **1** | 59 | 2.8E-02* | 67 | 6.6E-05** | 50 | 5.5E-01 |
| **V3** | **2** | 66 | 1.1E-03** | 61 | 4.5E-03* | 58 | 5.0E-02* |
| | **3** | 54 | 2.2E-01 | 59 | 2.6E-02* | 52 | 3.5E-01 |
| | **4** | 66 | 2.0E-03** | 69 | 7.9E-04** | 53 | 2.9E-01 |
| | **0** | 86 | 6.6E-05** | 90 | 6.6E-05** | 66 | 1.1E-03** |
| | **1** | 89 | 6.6E-05** | 92 | 6.6E-05** | 67 | 1.3E-04** |
| **V3A** | **2** | 61 | 2.6E-02* | 68 | 3.3E-04** | 51 | 4.3E-01 |
| | **3** | 70 | 1.3E-04** | 74 | 6.6E-05** | 55 | 1.4E-01 |
| | **4** | 75 | 1.3E-04** | 83 | 6.6E-05** | 57 | 3.5E-02* |
| | **0** | 66 | 2.0E-03** | 68 | 4.6E-04** | 66 | 5.3E-04** |
| | **1** | 65 | 3.6E-03* | 60 | 1.9E-02* | 47 | 7.2E-01 |
| **V4** | **2** | 58 | 3.4E-02* | 64 | 9.2E-04** | 53 | 2.7E-01 |
| | **3** | 49 | 5.9E-01 | 54 | 1.9E-01 | 52 | 3.2E-01 |
| | **4** | 51 | 4.2E-01 | 57 | 5.1E-02 | 59 | 2.6E-02* |
| | **0** | 89 | 6.6E-05** | 77 | 6.6E-05** | 54 | 2.0E-01 |
| | **1** | 70 | 1.3E-04** | 79 | 6.6E-05** | 54 | 1.8E-01 |
| **V5** | **2** | 80 | 6.6E-05** | 79 | 6.6E-05** | 49 | 6.2E-01 |
| | **3** | 81 | 6.6E-05** | 73 | 6.6E-05** | 53 | 2.5E-01 |
| | **4** | 74 | 6.6E-05** | 69 | 5.3E-04** | 50 | 5.6E-01 |

* denotes significance at uncorrected $p$ = 0.05.
** denotes significance at Bonferroni-corrected $p$ = 2.8E-03.
IP: In-phase stimulus. CP: Counter-phase stimulus. NM: Non-motion stimulus.

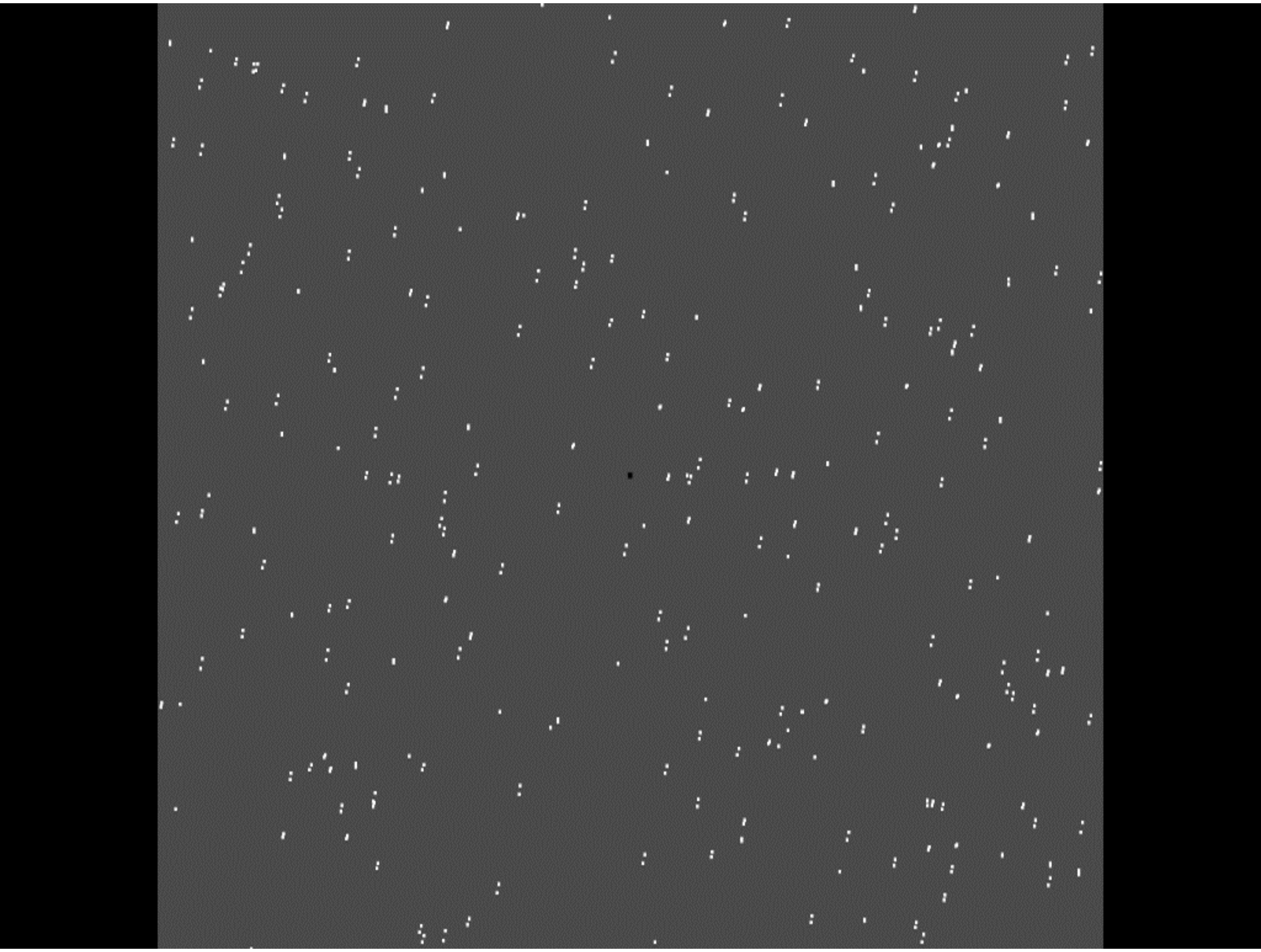

**Video A** . Example video of IP, CP, and NM trials composing a full experimental stimulus block. During the experimental scanning sessions, a block contained 6 trials of the same paired-dot stimulus condition, all exhibiting Glass patterns that were randomly oriented 15 degrees clockwise or counterclockwise from the block's overall cardinal direction. Trials were 1.1 seconds long, and participants indicated whether the Glass pattern was clockwise or counterclockwise. In the video, three vertical IP trials are presented, oriented counterclockwise, counterclockwise, and clockwise, respectively. Three vertical CP trials are then presented, also oriented counterclockwise, counterclockwise, and clockwise, respectively. Finally, three vertical NM trials are presented, oriented clockwise, counterclockwise, and counterclockwise.
